# Drug Reaction With Eosinophilia and Systemic Symptoms-Associated Perimyocarditis After Initiation of Anti-tuberculosis Therapy: A Case Report

**DOI:** 10.7759/cureus.37399

**Published:** 2023-04-10

**Authors:** Darren Kong, Keshav Dixit, Swiri Konje, Kruti Gandhi, Sidra Salman, Errol Moras, Vikram Agarwal

**Affiliations:** 1 Medicine, Mount Sinai Morningside, New York, USA

**Keywords:** tuberculosis, perimyocarditis, dress syndrome, drug reaction with eosinophilia and systemic symptoms, myopericarditis, anti-tuberculosis drugs, anti-tuberculosis medications, drug reaction with eosinophilia and systemic symptoms (dress) syndrome, drug reaction with eosinophilia and systemic symptoms (dress)

## Abstract

A 34-year-old female who was recently placed on anti-tuberculosis medication with rifampin, isoniazid, pyrazinamide, and levofloxacin therapy for suspected tuberculosis reinfection presented with subjective fevers, rash, and generalized fatigue. Labs showed signs of end-organ damage with eosinophilia and leukocytosis. One day later, the patient became hypotensive with a worsening fever, and an electrocardiogram showed new diffuse ST segment elevations with an elevated troponin. An echocardiogram revealed a reduction in ejection fraction with diffuse hypokinesis, and cardiac magnetic resonance imaging (MRI) showed circumferential myocardial edema with subepicardial and pericardial inflammation.

Prompt diagnosis of drug reaction with eosinophilia and systemic symptoms (DRESS) syndrome using the European Registry of Severe Cutaneous Adverse Reaction (RegiSCAR) criteria and discontinuation of therapy was initiated. Due to the hemodynamic instability of the patient, the patient was started on systemic corticosteroids and cyclosporine, with the improvement of her symptoms and rash. A skin biopsy was performed, which revealed perivascular lymphocytic dermatitis, consistent with DRESS syndrome. As the patient’s ejection fraction improved spontaneously with corticosteroids, the patient was discharged with oral corticosteroids, and a repeat echocardiogram showed full recovery of ejection fraction.

Perimyocarditis is a rare complication of DRESS syndrome that is associated with degranulation and the release of cytotoxic agents into myocardial cells. Early discontinuation of offending agents and initiation of corticosteroids are essential to rapid recovery of ejection fraction and improved clinical outcomes. Multimodality imaging, including MRI, should be used to confirm perimyocardial involvement and guide the necessity for mechanical support or transplant. Further research should be on the mortality of DRESS syndrome with and without myocardial involvement, with an increased emphasis on cardiac evaluation in DRESS syndrome.

## Introduction

Drug reaction with eosinophilia and systemic symptoms (DRESS) syndrome is a drug-induced hypersensitivity T-cell mediated reaction, which can occur due to a variety of medications, more commonly anticonvulsants and sulfonamides [1]. The pathogenesis is not yet fully understood but is hypothesized to be caused by an accumulation of reactive metabolites [1,2]. The clinical presentation, often developing two to six weeks after treatment initiation, is very diverse with multi-systemic involvement [1-3]. Patients most commonly present with fevers, skin findings (classically a diffuse morbilliform eruption), lymphadenopathy, and/or hematological abnormalities (eosinophilia, lymphocytosis, and neutrophilia). Skin findings are seen in almost 99% of patients, while fevers are the most common sign of disease [3].

There is no established standard criterion for diagnosing DRESS syndrome, and given the diverse presentation, diagnosing DRESS syndrome can be quite challenging in some patients. Two scoring systems often utilized to combat this challenge are the European Registry of Severe Cutaneous Adverse Reaction (RegiSCAR) criteria and the Japanese Research Committee on Severe Cutaneous Adverse Reaction (J-SCAR) criteria [1]. A recent retrospective study evaluating 390 patients concluded that the J-SCAR criteria failed to diagnose a significant proportion of DRESS syndrome cases and recommended the RegiSCAR scoring system [4].

Apart from anticonvulsants and sulfonamides, several antimicrobials have also been associated with DRESS syndrome in the past. There have been several case reports of DRESS syndrome developing in patients following the initiation of RIPE (rifampin, isoniazid, pyrazinamide, fluoroquinolone) therapy [5-10]. In all of these cases, patients presented with rash and/or fevers along with eosinophilia. All of these patients showed improvement following cessation of anti-tuberculosis therapy and early initiation of glucocorticoids.

Unfortunately, DRESS syndrome is also associated with several long-term complications such as type I diabetes mellitus, thyroid dysfunction, autoimmune hemolytic anemia, and multiple organ failure with and without decompensation [11]. Cardiac involvement is a rare but potentially fatal sequela of DRESS syndrome. DRESS syndrome can often precipitate the development of perimyocarditis in such patients and while rare, post-DRESS perimyocarditis can be fatal. There have been a few such cases reported, all of which highlight the importance of initiating corticosteroid therapy for such patients [12,13].

## Case presentation

A 34-year-old female immigrant from the Philippines with a history of latent tuberculosis, Lyme disease, and Crohn’s disease on infliximab treatment presented to our hospital with a fever and mild rash. One month prior, the patient was hospitalized for shortness of breath and was found to have a large right pleural effusion on chest X-ray, for which a chest tube was placed and a pleural fluid analysis was sent. Due to a concern for reactivated tuberculosis from infliximab immunosuppression, the patient was placed on a three-month course of rifampin, isoniazid, pyrazinamide, and levofloxacin and subsequently discharged. Several weeks later, she began noticing a rash that started on her face, which eventually spread to her chest, back, and arms. The patient also started to experience intermittent fevers, abdominal pain, lightheadedness, and decreased appetite, prompting her to visit our hospital.

**Figure 1:**
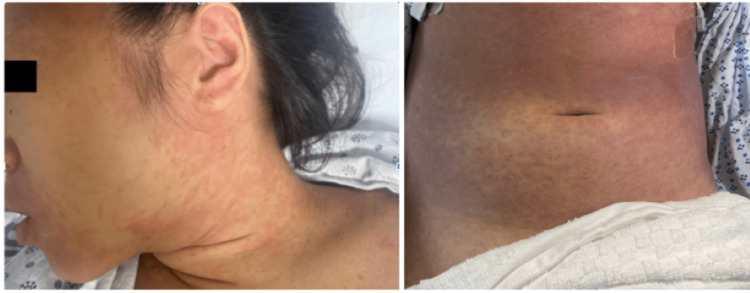
Patient with a diffuse, erythematous, morbilliform rash of the face, trunk, and back.

In the emergency department, the patient was alert and oriented but uncomfortable. Vitals on presentation were a blood pressure of 80/52 mmHg, heart rate of 131 beats per minute, temperature of 104.5°F, respirations at 22 breaths per minute, and oxygen saturation at 99% on room air. Physical exam was remarkable for dry mucosa, tenderness to the right upper quadrant and epigastric region, decreased breath sounds on the right lung base, and diffuse, pruritic, morbilliform rash on the face, chest, back, abdomen, and arms with focal peri-oral rash (Figure 1).

Laboratory findings were significant for transaminitis of 598/1637 IU/L, lactate at 4.2 mmol/L, and alkaline phosphatase (ALP) at 142 IU/L. Bilirubin and lipase levels were unremarkable. White blood cell count was unremarkable at 7.3 K/uL; however, the cell differential showed an eosinophil percentage at 5.7% (normal range: 0.0-5.0%) with an absolute eosinophil count of 0.7 (normal range: 0.0-0.6 K/uL). Antinuclear antibodies (ANA) were also positive as well. An initial two-dimensional transthoracic echocardiogram in the emergency department revealed no significant flow abnormalities and a normal left ventricular ejection fraction of 65%. Initial diagnosis of DRESS syndrome was used with the RegiSCAR inclusion criteria.

Other investigatory findings that were ordered included Lyme total antibodies, *Ehrlichia chaffeensis* antibodies, HIV-1 viral load, cytomegalovirus (CMV) polymerase chain reaction (PCR), CMV antibodies, *Mycoplasma* antibodies, coronavirus disease 2019 (COVID-19) PCR, stool ova and parasites, and hepatitis panel. Hepatic labs, including ceruloplasmin, ferritin, anti-smooth muscle antibodies, alpha-1-antitrypsin, and anti-mitochondrial antibodies, were negative. Rheumatologic labs, including anti-centromere, anti-chromatin, anti-double-stranded DNA, anti-Jo1, anti-scleroderma-70, anti-ribonuclear protein, anti-Sjögren’s syndrome antigen A (anti-SSA), anti-Sjögren's syndrome antigen B (anti-SSB), and anti-Smith antibodies, as well as perinuclear antineutrophil cytoplasmic antibody (p-ANCA) and cytoplasmic antineutrophil cytoplasmic antibody (c-ANCA) vasculitis panels, were negative. Urine toxicology, blood acetaminophen, and salicylate were also unremarkable.

**Figure 2:**
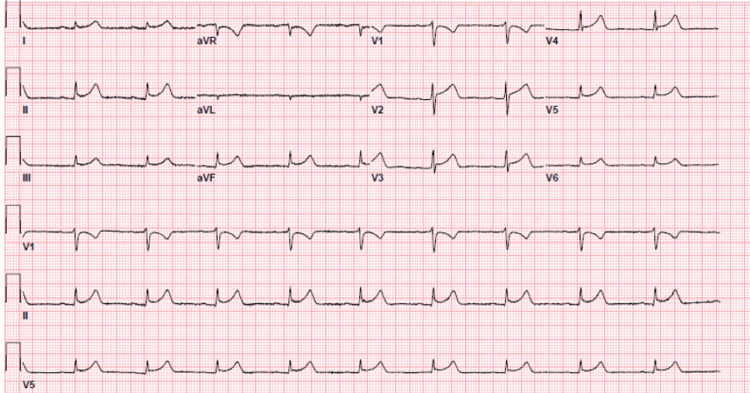
Electrocardiogram (EKG) findings showing diffuse ST segment elevations with concern for perimyocarditis.

The patient was initially treated with fluid resuscitation, intravenous (IV) diphenhydramine, and antibiotics with piperacillin-tazobactam and vancomycin. Despite these interventions, the patient’s blood pressure remained unstable. Liver function tests including aspartate aminotransferase (AST)/alanine aminotransferase (ALT) continued to rise at 1031/2808 IU/L, respectively, and the patient’s creatinine rose to 1.27 mg/dL. Electrocardiogram (EKG) at the time showed diffuse ST elevations in the inferior and anterolateral leads (Figure 2). Point-of-care ultrasound at the bedside revealed decreased systolic motion of the left ventricle, with an elevated troponin of 0.095 ng/mL. Given the patient’s hemodynamic instability and signs of multi-organ failure, the patient was transferred to the medical intensive care unit (ICU) for vasopressor support with norepinephrine.

**Figure 3:**
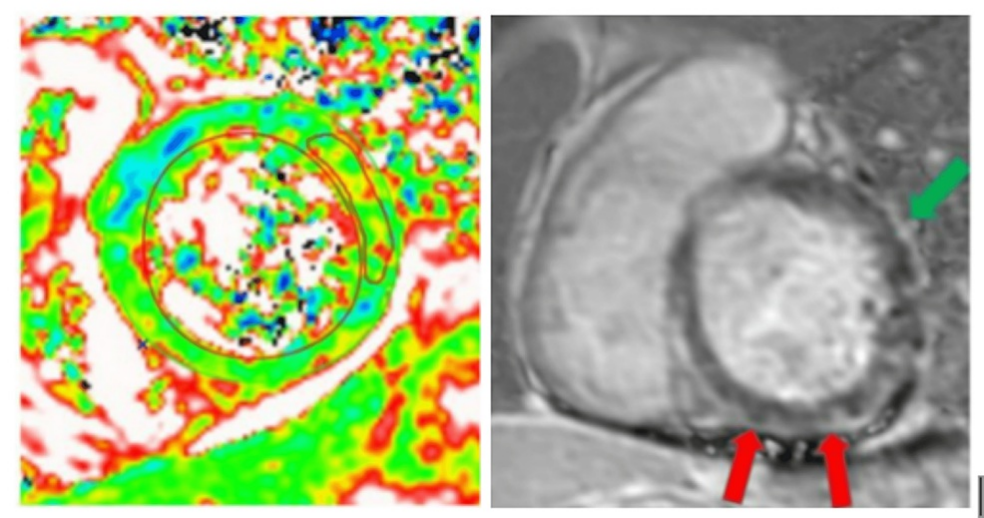
Left: Cardiac MRI displaying myocardial edema on T2-weighted images - the localized value in the ROI (region of interest) in 68 msec (normal < 60 msec). Right: Circumferential subepicardial (red arrow) and pericardial (green arrow) late gadolinium enhancement (LGE) with associated myocardial edema and acute pericarditis.

While in the medical ICU, the patient was started on systemic corticosteroids at a dose of 1 mg/kg daily and cyclosporine for the management of presumed DRESS syndrome. Tuberculosis coverage was replaced with doxycycline. Repeat transthoracic echocardiogram in the ICU showed inferolateral wall akinesis, inferior wall akinesis, left atrial dilation, and moderate left ventricular systolic dysfunction with an ejection fraction of 40%. Troponins continued to rise at 5.171 ng/mL. Due to concerns for new regional wall motion abnormality, cardiac computed tomography (CT) angiography was performed, which revealed a calcium score of 0 and normal coronaries. Cardiac magnetic resonance imaging (MRI) showed almost circumferential subepicardial late gadolinium enhancement (LGE) involving predominantly all the basal and mid-left ventricular segments, with concomitant patchy myocardial edema and associated pericardial inflammation and fibrosis, consistent with acute perimyocarditis (Figure 3).

**Figure 4:**
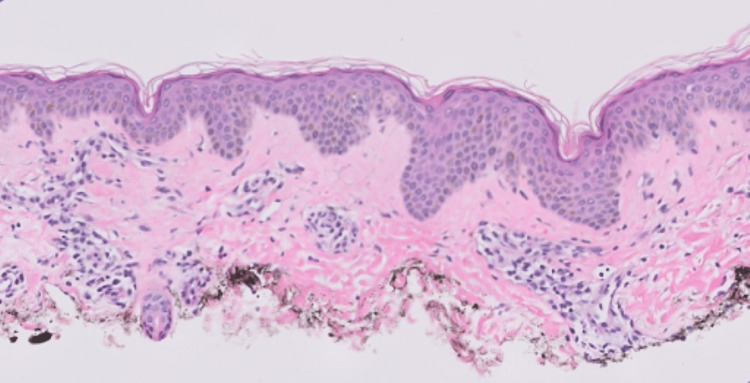
Skin biopsy with superficial perivascular lymphocytic dermatitis, consistent with DRESS syndrome. DRESS: drug reaction with eosinophilia and systemic symptoms.

The patient’s pressures began to improve and norepinephrine was discontinued. Acid-fast bacillus respiratory cultures were repeatedly negative throughout her ICU stay. She was transferred to the general medicine service floors, transitioned to oral corticosteroids, and weaned off of cyclosporine. Repeat transthoracic echocardiogram prior to discharge showed recovery of ejection fraction to 55%. A skin biopsy of the rash was obtained, which revealed superficial perivascular lymphocytic dermatitis, consistent with DRESS syndrome (Figure 4).

**Figure 5:**
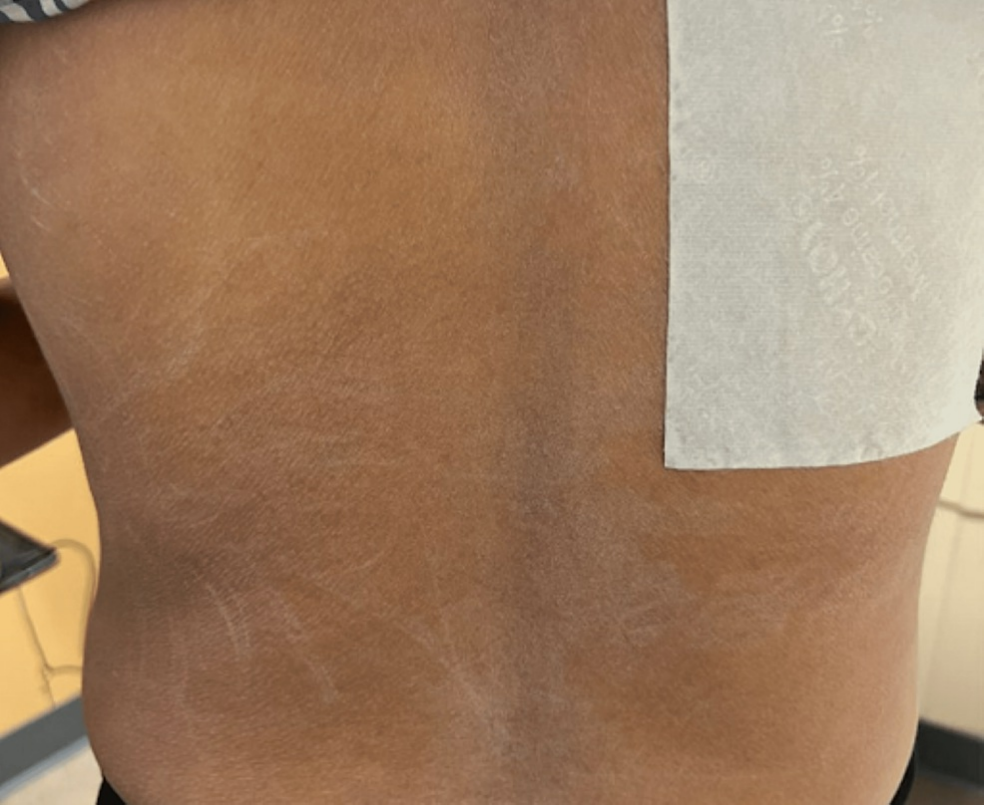
Skin exam of the back and trunk with the resolution of rash after outpatient corticosteroid use.

The patient remained hemodynamically stable and was subsequently discharged with close follow-up with our cardiology, dermatology, and pulmonology services. Outpatient testing and follow-up visits showed resolution of the patient’s transaminitis, normal troponins, ejection fraction of 65%, and near-resolution of the patient’s rash (Figure 5).

## Discussion

Diagnosis of DRESS syndrome was made using the RegiSCAR criteria [4,14]. Based on the criteria, the patient had an eosinophilia of 1.8 x 10^9^/L, skin rash with purpura and infiltration over >50% of body surface area, multi-organ involvement of the heart and liver, and exclusion of other causes, which indicates RegiSCAR score of 7, definitive for the diagnosis of DRESS syndrome (Table 1).

**Table 1:** RegiSCAR criteria. A score of <2 is not suggestive, 2-3 is possible, 4-5 is probable, and 5> is definitive for DRESS syndrome [14]. RegiSCAR: European Registry of Severe Cutaneous Adverse Reaction; DRESS: drug reaction with eosinophilia and systemic symptoms; ANA: antinuclear antibody; BSA: body surface area; HAV: hepatitis A virus; HBV: hepatitis B virus; HCV: hepatitis C virus; N: no; U: unknown; Y: yes; WBC: white blood cell.

Clinical assessments	Score			Comments
	-1	0	1	
Fever ≧ 38.5°C	N/U	Y		
Enlarged lymph nodes		N/U	Y	>1 cm and ≧ 2 different areas
Eosinophilia ≧ 0.7 x 10^9^/L or ≧ 10% increase if WBC < 4.0 x 10^9^/L		N/U	Y	Score 2, when ≧1.5 x 10^9^/L or ≧20% if WBC < 4.0 x 10^9^/L
Atypical lymphocytosis		N/U	Y	
Skin rash with >50% extension of BSA		N/U	Y	Rash suggesting DRESS: ≧2 symptoms: purpuric lesions (other than legs), infiltration, facial edema, psoriasiform desquamation
Skin rash suggestive of DRESS	N	U	Y	
Skin biopsy suggesting DRESS	N	Y/U		
Organ Involvement		N	Y	Score 1 for each organ involvement, maximal score 2
Rash resolution ≧ 15 days	N/U	Y		
Excluding other causes		N/U	Y	Score 1 if three tests of the following tests were performed and all were negative: HAV, HBV, HCV, *Mycoplasma*, chlamydia, ANA, blood culture

Current treatment of DRESS syndrome is primarily discontinuation of the offending agent, which was likely due to the initiation of anti-tuberculosis therapy. In cases of severe systemic disease, corticosteroids in conjunction with either cyclosporine and/or intravenous immunoglobulin (IVIG) have been shown to limit inflammation and degranulation of eosinophils.

While DRESS syndrome most commonly affects the liver and kidneys, perimyocardial involvement is not well documented. The pathophysiology for myopericarditis in the setting of DRESS syndrome is unclear. Thongsri et al. proposed that cardiac damage was the result of eosinophilic invasion and degranulation, leading to subsequent fibrotic scarring of the endomyocardium [12]. Deposits of major basic protein (MBP), eosinophil cationic protein (ECP), and eosinophil peroxidase (EPO), which is known to induce apoptosis and necrosis, were all found in patients with hypereosinophilic myocarditis. The extent of damage and cardiotoxicity is determined by the duration and degree of eosinophilia. In addition, endomyocardial biopsy in the setting of DRESS syndrome has shown eosinophilic infiltrate and myocardial necrosis [15].

When there is cardiac involvement from DRESS syndrome, eosinophils infiltrate the myocardium. Eosinophils contain several specific granules such as MBP and cationic protein, which are cardio-toxic agents. Once released, these proteins can lead to changes in the myocardial tissue, often causing cell necrosis and apoptosis to occur [12]. The damage to myocardial tissue upon the release of such granules varies, and thus it is imperative to evaluate these patients with ECGs and echocardiograms [12,16].

The mainstay treatment of DRESS-associated perimyocarditis is to discontinue the offending agent and start high-dose corticosteroids. Additionally, patients should also be started on guideline-directed medical therapy if there is a reduction in ejection fraction. Even though these patients are at increased risk for developing ventricular arrhythmias as both ventricles are generally affected, it is not recommended to start anti-arrhythmic therapy. Rather, it is crucial to monitor these patients closely and treat any arrhythmias that arise accordingly.

Because it is common for patients to experience a relapse of DRESS symptoms, steroids should be tapered slowly over several weeks to months to allow patients to undergo sufficient immunosuppression. There is no current consensus on steroid dosage or duration of taper; however, if recurrence occurs during the steroid taper, steroid-sparing agents such as cyclosporine, IVIG, or mycophenolate should be considered. In addition to high-dose corticosteroids, IVIG and/or mycophenolate mofetil have also been used for patients with fulminant myocarditis, which has high mortality rates. To monitor heart function, these patients should routinely be evaluated with echocardiograms, and among patients with refractory heart failure, ventricular assist device implantation, intra-aortic balloon pumping, and/or ECMO support should be considered.

Early diagnosis with the initiation of treatment and close follow-up will be essential as advanced forms of myocarditis, such as acute necrotizing eosinophilic myocarditis (ANEM), have a greater than 50% mortality rate with a median survival of only three to four days [16]. In a systematic review by Radovanovic et al., shorter latency between drug exposure and symptom onset and older age correlated with increased mortality [17]. With regard to antimicrobial-associated DRESS, the highest mortality rate is associated with tetracyclines and anti-tuberculosis antibiotics [17,18]. Radovanovic et al.’s study also showed over half of the DRESS syndrome patients with cardiac involvement presented with dyspnea and chest pain [16]. These patients should warrant further cardiac evaluation, including initial cardiac biomarkers and an EKG.

## Conclusions

Further research should be toward greater emphasis on cardiac evaluation in DRESS syndrome and the role of multimodality cardiac imaging to confirm perimyocardial involvement and guide management strategies. In addition to the assessment of the biventricular size and systolic function using an echocardiogram, myocardial tissue characterization using cardiac MRI can help identify and quantify the extent of perimyocardial inflammation, which may prompt the necessity for closer monitoring, and identify the need for more invasive measures, including mechanical support or transplant, at an early stage. In our case report, the severe reduction in ejection fraction and evidence of extensive perimyocardial involvement prompted our team to escalate this patient to the ICU and use both systemic corticosteroids and cyclosporine for drug therapy.
